# Physical therapist management and coordination of care to prevent pathological hip fracture from metastatic disease: a case report

**DOI:** 10.3389/fresc.2024.1384782

**Published:** 2024-09-19

**Authors:** Athena Manzino, Christopher Wilson

**Affiliations:** ^1^ Physical Medicine and Rehabilitation, Beaumont Health, Royal Oak, MI, United States; ^2^ Physical Therapy Program, Oakland University, Rochester, MI, United States

**Keywords:** breast cancer, intervention, skeletal-related event, surgical fixation, rehabilitation, physical therapy, oncology

## Abstract

**Background:**

Bone metastases are common in patients with progressive cancer and often present in long bones, leading to adverse events such as pathologic fractures. In the acute care setting, physical therapists (PTs) may be the initial providers who identify symptoms associated with fracture risk and communicate concerns to help prevent such adverse events.

**Case description:**

A 39-year-old female patient with metastatic breast cancer was admitted to the hospital due to uncontrolled pain. She had a history of bone metastases to the left femur; however, no restrictions or precautions were noted during the initial PT examination. During this initial PT examination, she reported worsening hip pain with weight-bearing activities.

**Outcomes:**

With the PT's recognition of red flag symptoms, an MRI was completed, which revealed extensive metastatic disease in her left femur with concern for an imminent fracture; as a result, prophylactic fixation was performed. Her functional abilities improved after surgery and consistent therapeutic intervention, allowing her to achieve a level of independence sufficient to return home safely.

**Discussion and conclusion:**

This case demonstrates the successful identification of imminent fracture risk by a PT in a patient with metastatic breast cancer, as well as the therapeutic management that accompanied this process in the acute care setting.

## Introduction

The typical progression of breast cancer commonly results in metastatic bone disease (1, 2), with approximately 30%–40% of recurrence cases progressing to some form of bone metastases (3). This can lead to a variety of complications, commonly referred to as skeletal-related events (SREs). One of the more severe complications is pathological fractures of long bones, with the femur being at high risk due to the force transmission that occurs during mobility and transfers. It is well known that pathological fractures are associated with decreased survival rates (4) and increased hospitalizations in patients with breast cancer (5).

Scales currently exist as evaluation tools to quantify the level of fracture risk in long bones, with one of the most commonly used being the Mirels scale (6). By considering the location of the metastases, patient-reported pain levels, and the size and type of metastases based on imaging, the Mirels scoring system evaluates the risk of sustaining a pathological fracture in a long bone with known metastatic disease (6–9). In addition, one of the most crucial evaluation components for a physical therapist (PT) working with cancer patients in the acute care setting is the observation of pain during weight-bearing activities and its link to potential undiagnosed metastatic bone disease and risk of SRE (10).

Despite the risk of pathologic bone fracture, exercise is considered important for individuals with bone metastases; however, careful interdisciplinary communication and shared decision-making are essential in these cases (10). In addition, bone mineralization is important to address through adequate mineral absorption (e.g., calcium, vitamin D) through diet or nutritional supplementation, as well as pharmacologic management through medication (e.g., bisphosphonates, denosumab) to slow bone reabsorption (11). The purpose of this case report was to describe a PT's involvement in the diagnostic and decision-making process for a metastatic femoral lesion and the perioperative care for a prophylactic hip fixation secondary to bone metastases in a patient with breast cancer in the acute care setting. This case report was written utilizing the CARE guidelines (12).

## Case description

A 39-year-old woman was hospitalized 25 months after being diagnosed with stage III breast cancer. She was hospitalized for 41 days, during which she received 22 sessions of physical therapy. The patient signed an informed consent for this case report, and the Beaumont Institutional Review Board determined that review and approval were not warranted, as this is a retrospective case report.

The patient, a 39-year-old woman, was diagnosed with T3N1M0 breast cancer 2 years prior to the hospital admission described in this case report (Table 1). Histological assessment revealed it to be triple-negative invasive ductal carcinoma. She initially sought evaluation following the onset of a lump in her left breast. Her past medical history was limited to asthma, and her BMI upon admission was 32.7; she was otherwise healthy, active, working full-time, and a non-smoker. She resided in a one-story home with her husband and three young boys, with three entry steps and no handrails.

**Table 1:** Timeline of key events prior to hospitalization.

Time in relation to hospitalization (negative value is prior to admission)	Key event
−25 months	Diagnosed with triple-negative breast cancer.
−20 months	Completed neoadjuvant chemotherapy consisting of doxorubicin hydrochloride (Adriamycin), cyclophosphamide (AC), and weekly paclitaxel (Taxol).
−18 months	Underwent double mastectomy with bilateral tissue expander placement and left axillary lymph node dissection.
−17 months	Admitted to the hospital for fever and right breast pain, found to have post-surgical methicillin-susceptible *Staphylococcus aureus* (MSSA) infection, which was treated successfully with antibiotics; underwent surgical removal of right tissue expander.
−16 months	Began post-operative chemotherapy consisting of carboplatin (Paraplatin).
−14 months	Participated in outpatient physical therapy for bilateral upper extremity lymphedema.
−13 months	Began radiation therapy.
−9 months	Underwent surgical replacement of right tissue expander.
−8 months	Underwent surgical revision of right tissue expander due to poor healing.
−3 months	Underwent surgical bilateral breast implantation.
−2 months	Participated in outpatient physical therapy for lymphedema when referred by a physical therapist for an MRI of the left shoulder due to ongoing pain.
−1 month	MRI of the left shoulder showed possible metastatic osseous disease.
−17 days	A new PET scan showed extensive osseous and liver metastases.
−15 days	Brain MRI showed left caudate lobe metastasis.
−11 days	A liver biopsy confirmed adenocarcinoma of breast origin, and the patient was prescribed pain medication with immediate reports of poor tolerance.

Following her initial diagnosis, the patient underwent neoadjuvant chemotherapy, followed by bilateral mastectomies with tissue expanders and a left axillary lymph node dissection 6 months after her initial diagnosis. Post-surgical pathological examination did not detect disease in the right breast, while residual invasive ductal carcinoma and left axillary sentinel lymph node involvement were detected in the left breast. The patient was then treated with adjuvant chemotherapy and subsequent radiation therapy. Due to an infection in the right breast tissue expander, she required multiple surgical revisions, eventually undergoing bilateral implant surgery 15 months after her initial mastectomies.

**Figure 1:**
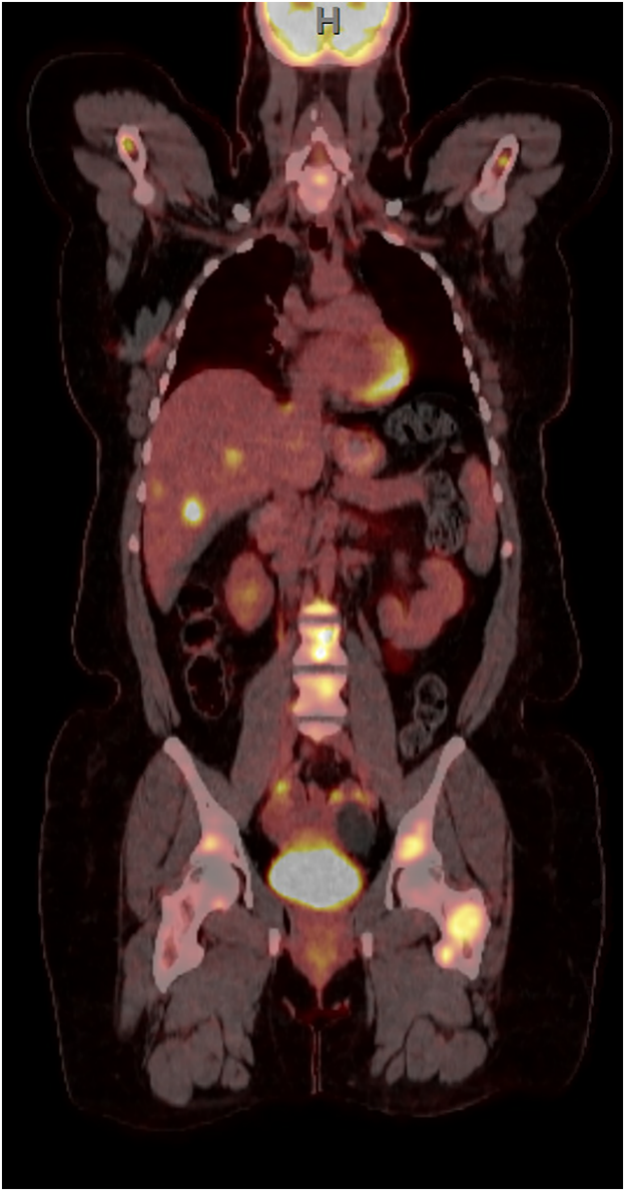
PET scan showing widespread metastatic disease.

In the following months, the patient was treated for upper extremity lymphedema in an outpatient physical therapy setting; during this time, she began to experience pain in her left upper extremity. Magnetic resonance imaging (MRI) showed new osseous metastases in the left shoulder, and a positron emission tomography (PET) scan identified metastases in the right mandible, multiple thoracic spine vertebrae, pelvis, right clavicle, right scapula, bilateral ribs, sternum, bilateral humeri, and the left femur, notably in the intertrochanteric region (Figure 1).

The patient was admitted to the hospital for pain control 25 months after her initial diagnosis. A PT consultation was initiated 4 days after admission, and she also received concurrent occupational therapy during her stay. Notably, within the medical record, there were no mobility restrictions, the patient was full weight-bearing, and there was no substantive mention of the femoral lesion beyond its initial identification on the PET scan. The patient's physical therapy interventions consisted of four distinct phases, each involving different treatment intentions and clinical approaches; these phases are described in separate sections of this manuscript (Table 2). See Table 3 for a timeline of key events for this patient during her hospitalization.

**Table 2:** Phases of rehabilitation.

Phase	1	2	3	4
Visit number	1–4	5–6	7–10	11–22
Intervention approach	Evaluation and initial treatment	After intramedullary nail placement	Progressive functional mobility	Preparing for hospital discharge to home

**Table 3:** Timeline of key events during hospitalization.

Time in relation to admission	Key event
Day 1	Admission to hospital for poor tolerance to currently prescribed pain medication.
Day 5/visit 1 (phase 1)	Initial physical therapy evaluation was conducted. The patient reported pain in her back and left hip with movement and weight bearing. The PT communicated with the attending physician about concerns about left hip stability.
Day 8/visit 2 (phase 1)	Educational PT session was provided due to the patient’s reports of generalized fatigue and global pain preventing mobility at that time.
Day 9/visit 3 (phase 1)	The patient ambulated 20 feet with a rolling walker before requiring cessation of the session due to reports of increasing left hip and shoulder pain with mobility.
Day 13/visit 4 (phase 1)	The patient attempted one step with the rolling walker before reporting left hip pain and was immediately instructed by the PT to return to bed. She then reported feeling as though her left hip was “giving out” during ambulation the previous day. PT contacted the nurse and attending physician regarding ongoing concerns due to increasing pain and instability with weight-bearing activities.
Day 14	Weight-bearing status did not change, and the PT contacted the physician with a recommendation for imaging. An x-ray was performed, and the results were negative.
Day 15	MRI was performed.
Day 16	MRI of the left hip was performed and showed advanced metastatic disease within the intertrochanteric region. The patient was now placed on NWB restrictions for the left hip.
Day 17	The patient underwent left hip prophylactic surgical fixation with intramedullary nail insertion.
Day 18/visit 5 (phase 2)	The patient was re-evaluated during the physical therapy session and noted to have high pain levels preventing mobility and tolerance to activity limited to light left lower extremity PROM in a very limited range.
Day 19/visit 6 (phase 2)	The patient was able to tolerate transferring to sitting at the EOB and minimal therapeutic exercises, limited by left lower extremity pain.
Day 21/visit 7 (phase 3)	The patient was able to stand at the EOB with a rolling walker and moderate assistance, but further mobility was limited due to pain.
Day 22/visit 8 (phase 3)	The patient was able to tolerate small distance ambulation to the bedside commode with 25% weight bearing through the left lower extremity.
Day 26/visit 9 (phase 3)	The patient ambulated 40 feet during the therapy session.
Day 28/visit 11 (phase 4)	The patient was able to perform three repetitions of sit-to-stand transfers during the 30-s sit-to-stand test.
Day 40/visit 21 (phase 4)	The patient ambulated 30 feet with handheld assistance.
Day 41/visit 22 (phase 4)	The patient ambulated 140 feet with physical therapy and was discharged from the hospital later that day.

At the initial examination, 5 days after hospital admission, the patient was referred to acute care physical therapy. She did not report hip pain except during weight-bearing activities and verbalized moderate left hip pain, rating it as 6 out of 10 on the numeric pain rating scale. A cycle of chemotherapy was started during this admission, and she also was treated with fentanyl and hydromorphone for pain relief.

### Phase 1: pre-surgery (visits 1–4)

At the initial evaluation, the patient demonstrated the ability to log roll and transition from supine to sitting with stand-by assist (SBA) using one bed rail, taking increased time, and requiring verbal cueing for proper form. She was able to maintain unsupported seated balance and reach outside of her base of support without upper extremity support. A sit-to-stand transfer at the edge of the bed (EOB) was performed without an assistive device with contact guard assist (CGA), and mild difficulty maintaining balance independently during dynamic movement. Ambulation was not possible at this time due to pain reported in the left hip and back upon standing, as well as dizziness. The Activity Measure for Post Acute Care (AM-PAC) 6-Clicks Basic Mobility form was utilized to assess activity limitations, and her score was recorded as 16/24 (6 = lowest mobility, 24 = highest mobility) (13, 14). Goals were created to focus on increasing her level of mobility to modified independent (Mod I) with bed mobility, transfers, gait for 50 feet with a rolling walker (RW), and stair negotiation of three steps, in addition to independence with a home exercise program (HEP). Following the initial treatment session, the therapist communicated with the attending physician, recommending an orthopedic consult or additional imaging due to patient-reported symptoms in the left hip region, which likely warranted further evaluation.

The patient was seen by a physical therapist 3 days after an educational visit (visit 2) due to hypertension that was outside the therapeutic range for out-of-bed (OOB) mobility; the patient was seen again for OOB mobility a few days later. At visit 3, the patient demonstrated bed mobility and sit-to-stand transfers with SBA and the use of an RW. The patient was able to ambulate 20 feet to the bedside chair with CGA/SBA and a RW; she demonstrated an antalgic gait pattern, decreased cadence, shorter step length, and a wide base of support with one rest break and reported 5/10 hip pain. Compared to the initial session, the patient demonstrated increased difficulty with seated and standing balance, requiring upper extremity support and showing a decreased ability to adapt to functional challenges. The patient reported increased pain in the left hip and scapular region, which limited further mobility during the session. The AM-PAC 6-Clicks score improved slightly to 17/24 due to gait improvements, although there was evidence of a decline in balance and decreased tolerance to lower extremity therapeutic exercises, as noted by fewer repetitions completed with a Borg exertion rating of 12–14/20. While the goals from the initial treatment session remained the same, the treatment sessions were focused on patient participation and tolerance and education to complete in-bed exercises independently throughout the day within symptom-tolerated limits.

In the next therapy session (visit 4), the patient reported an increase in left hip pain in the previous few days and verbalized a feeling of instability in the hip, describing it as if it were going to “give out.” The patient was able to perform bed mobility with Mod I and sit-to-stand transfers with CGA and an RW; however, she required more time due to substantial left hip pain that worsened with weight-bearing activities. During OOB mobility, the patient demonstrated one step forward and backward with the RW, but she required minimal assistance (Min A) from the PT and used substantial upper extremity support while stepping, demonstrating a severe antalgic gait. Based on this increasing evidence of potential structural dysfunction, the treatment session was ceased, and she was returned to bed by the therapist. Furthermore, pain prevented the completion of any open-chain therapeutic exercise during this visit. Her AM-PAC 6-Clicks score remained stable at 17/24, reflecting improvements in bed mobility despite decreased gait abilities. The patient was educated on initiating lower extremity exercises, and she was able to tolerate seated ankle pumps and long arc quads bilaterally for 10 repetitions, each with a Borg score of 12/20 at the end of the session.

Following visit 4, the PT determined that functional mobility was not in the patient's best interest until orthopedic stability could be re-established. Given the patient's medical history and disease staging, the likelihood of progressive hip bone metastases to the point of imminent pathological fracture warranted further imaging. The most recent PET scan, conducted 1 month prior, had already described global osseous metastases, including an increased signal at the intertrochanteric region of the left femur.

**Figure 2:**
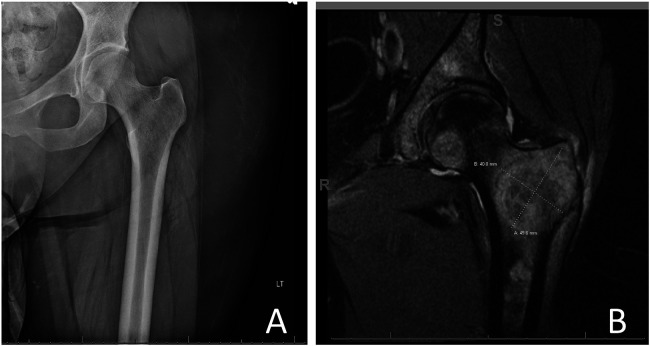
**(A)** Plain film x-ray of the left hip and **(B)** T1-weighted MRI of the left hip showing a large left hip lesion.

At this time, the PT contacted the physician again, as no apparent steps had been taken regarding these concerns despite mounting evidence to support that the patient's hip pain was of metastatic origin, raising concerns of an imminent SRE. Following this interdisciplinary communication, imaging of the left hip was ordered, and the initial X-ray appeared unremarkable (Figure 2A). However, a follow-up MRI showed extensive osseous metastatic disease with deep soft tissue edema surrounding the proximal left femoral intertrochanteric region (Figure 2B). Two days later, orders were initiated for non-weight bearing on the left lower extremity. Surgery was completed for prophylactic left hip fixation by intramedullary nail insertion, with the surgical report describing extensive metastatic disease within the intertrochanteric region but no evidence of pathologic fracture. After surgery, the patient was allowed to return to weight bearing as tolerated (WBAT), and physical therapy services were resumed post-operatively.

### Phase 2: after intramedullary nail placement (visits 5 and 6)

Phase 2 of therapy began with a re-evaluation 1 day after left hip surgical prophylactic fixation. Visit 5 started with the patient reporting improvements in pain from the previous day. However, she could only tolerate light range of motion exercises in a severely limited range at the left lower extremity while in a semi-Fowler position, and functional mobility was not examined due to pain. Her fatigue level on the Borg scale was rated at 11/20, and her AM-PAC 6-Clicks score decreased to 10/24.

At the start of visit 5, the patient's left hip was positioned in flexion. She was able to tolerate some improvements in decreased hip flexion through mechanical bed adjustments but was unable to achieve a neutral hip position. She expressed fatigue and discomfort throughout the entire left lower extremity upon completing 10 repetitions of ankle pumps. Substantial difficulty was observed with 10 repetitions of left hip abduction, and she could only abduct 1–2 inches from the starting position by passive range of motion (PROM) due to pain. In addition, she completed gluteal and quadriceps isometric exercises for 10 repetitions each, with decreased muscular activation noted and mild pain reported. Education was provided for in-bed exercises to be completed throughout the day to reduce stiffness in the left hip, with a goal of 10 repetitions of active range of motion (AROM) per hour, to tolerance.

During visit 6, the patient could transfer from supine to sitting at the EOB with moderate assistance (Mod A), needing support for eccentric control of the left lower extremity. Movement was noted to be slow and controlled due to pain, but she demonstrated safe movement patterns during transitional movements. Her static and dynamic sitting balance improved, allowing her to not require upper extremity support for reaching activities, but her AM-PAC 6-Clicks score remained 10/24. During this visit, she was able to tolerate left lower extremity exercises, including 20 ankle pumps, 3 short arc quads while sitting at the EOB, 10 repetitions of passive hip flexion in semi-Fowlers in a limited range, and 5 repetitions of left hip abduction PROM in semi-Fowlers. Improvements in range of motion and function were slow in subsequent sessions due to inadequate pain control and symptoms associated with the start of a new chemotherapy cycle.

### Phase 3: progressive functional mobility (visits 7–10)

Phase 3 began almost 1 week following surgery, with the initiation of bed mobility; the patient demonstrated an increased ability to move the left lower extremity independently throughout the session. She was able to roll and transition from supine to sitting with Min A from the therapist, especially for eccentric lowering of the left lower extremity. She performed sit-to-stand and a standing walking transfer to a bedside commode with Min-Mod A from the therapist and the use of an RW. Her AM-PAC 6-Clicks score improved to 12/24. By session 9, the patient was ambulating 40 feet with the RW and Min A while demonstrating an antalgic gait, a wide base of support, decreased step length, forward-flexed posture, and increased weight bearing through bilateral upper extremities. At this point, her AM-PAC 6-Clicks score improved to 16/24.

At this point, her pain was controlled by a patient-controlled analgesic pump, which she consistently used throughout the sessions. Functionally, emphasis was placed on bed mobility, standing, and progressive ambulation with an RW. Education on a HEP and answering the patient’s questions continued to be integral throughout this phase. As in previous visits, her rehabilitation potential remained good, and her support system remained intact. The goals for therapy remained the same as those established in the initial treatment session.

Active-assisted range of motion (AAROM) exercises were tolerated at the hip and knee with reports of decreased pain and stiffness. She continued to experience difficulty with left long arc quads when seated at the EOB, achieving five repetitions per session with slow movements and noted verbalizations of pain. In addition, despite being WBAT, the patient was only accepting a minimal amount of weight through her left lower extremity (∼toe touch weight bearing) during ambulation due to pain. She made improvements throughout this phase to 25% weight bearing and eventually to foot-flat weight bearing, with fewer verbal cues required.

### Phase 4: preparing for discharge (visits 11–22)

The patient's functional mobility continued to improve throughout the following visits in phase 4, leading to an ambulation distance of 140 feet with a RW and CGA, demonstrating a step-through pattern with an antalgic gait, decreased step length, decreased cadence, and decreased verbal cueing required. Her pain was well controlled at this time, with reported pain levels of 0–2/10 on a numeric pain rating scale, which allowed for more functional activity training during physical therapy.

As discharge approached, the focus of therapy sessions shifted to ensuing safety for home mobility, tolerance to OOB mobility, and weight-bearing exercise. Lower extremity exercises were implemented during rest breaks throughout the sessions and via a HEP. Her medical status and support system remained stable throughout this phase.

During this final phase, the patient was able to tolerate increased lower extremity exercises, including 10 repetitions of long arc quads, 8 half bridges, 10 repetitions of seated hip flexion, and AROM for all left lower extremity joints. Full weight bearing through the left leg was achieved, and she was even able to ambulate 15 feet with handheld assistance and no assistive device. Education was provided throughout the treatments for proper assistive device use, HEPs, and general post-surgical care.

## Outcomes

**Figure 3:**
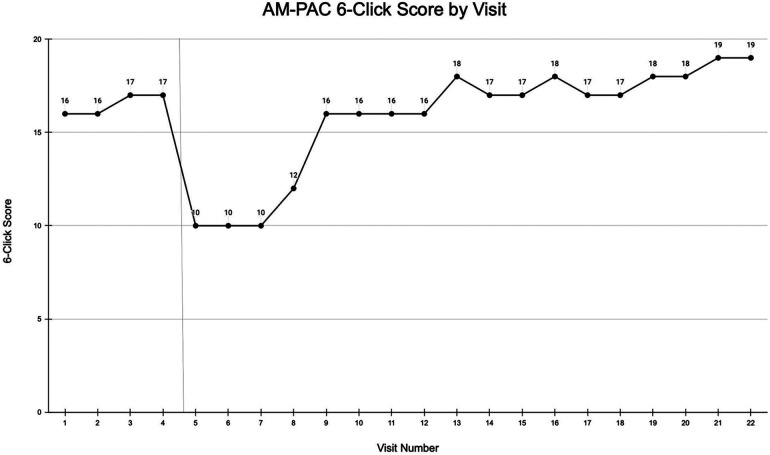
AM-PAC 6-Clicks scores by visit number. Prophylactic hip fixation surgery is indicated by a vertical line.

At discharge, the patient's functional mobility had improved to Mod I with bed mobility, SBA with transfers, and ambulation of 140 feet with a RW; she also demonstrated standing balance without upper extremity support with light reaching activities. Her AM-PAC 6-Clicks score continued to improve, reaching a final score of 18/24 during phase 4. Figure 3 depicts the AM-PAC 6-Clicks scores by visit number, with significant events noted.

Following discharge, the patient returned home with the RW and physical assistance from her spouse. She was instructed to continue with the HEP provided during her hospitalization and was prepared to participate in homecare physical therapy. Post-discharge goals included continuing to increase strength and range of motion, with an emphasis on activity tolerance and the hope of resuming ambulation without an assistive device.

## Discussion

The patient in this case was a 39-year-old woman with triple-negative breast cancer who, at the time of evaluation, presented with moderate hip pain. Over the course of her initial physical therapy treatments, the patient's hip pain increased, suggesting an increased likelihood of an SRE necessitating prophylactic internal fixation. Following a 41-day hospital stay, she made significant improvements in functional mobility for a safe discharge home. Most importantly, the PT's recognition of symptoms of an impending fracture and advocacy for further assessment and imaging likely prevented a pathological fracture and the medical complications that accompany it.

This case report provides several unique contributions to the scientific literature, including providing a descriptive clinical presentation of metastatic hip pain, highlighting the importance of proactive, collaborative interdisciplinary care, and providing detailed interventions and outcomes for clinicians to consider when encountering similar patients.

Physical therapists are often the first providers to perform functional evaluations for patients in acute care, utilizing a variety of tools such as patient-reported pain, strength, range of motion, and functional mobility to assess prognosis and guide treatment planning. Vigilance in identifying the onset of new or worsening symptoms can lead to differential diagnoses in the acute care setting (15), making communication between PTs and physician colleagues critical in evaluating risk and determining whether further examination is required. Evidence demonstrates that interdisciplinary collaboration helps to bridge the gap between patients and providers, specifically referring to monitoring patient-reported symptoms and ensuring safety (16).

It is common in advanced cancer to have multiple metastases, frequently affecting the bones, especially in the breast cancer population (17). It is well known that some bones, such as the femur, are more likely to develop metastases, which can lead to instability, pain, and SREs (18). Recent literature supports the use of exercise for patients with known bone metastases, emphasizing the importance of expert prescription and monitoring by PTs (19–21). However, there is limited education and literature surrounding the ability of PTs to identify when these metastases have progressed to the point of potential SREs, especially in the acute care setting.

Physical therapists are trained to identify symptoms and distinguish between differential diagnoses of hip pain with regard to orthopedic conditions, as there is much research to support this knowledge; however, there are likely opportunities for improved education on cancer-related causes of hip pain, including metastatic disease. Current evidence is limited to identifying cancer-related impairments that may require further assessment or imaging (22), despite the strong importance of early recognition of pain in the presence of metastatic disease as a red flag symptom needing referral.

As this is a case report, conclusions and generalizability are limited, and experimental clinical trials are needed to further explore this area of study. Although the AM-PAC 6-Clicks is a quick and effective measure of discharge recommendations based on functional mobility in the acute care setting, this measure has not been definitively tested for validity in the oncology population (23). Additional oncology-specific outcome measures may have been useful in quantifying other domains of health and quality of life. Although commonly used in post-surgical orthopedic care, the use of cryotherapy (e.g., ice packs) remains controversial in cancer care (24). However, given the extent of this patient's metastatic disease, there was likely an opportunity to use cryotherapy to reduce post-operative swelling and pain.

There is a paucity of evidence regarding the involvement of PTs in identifying risk factors for SREs from bone metastases, especially in the use of evidence-based assessment scales such as the Mirels scale (7). In addition, as PTs are not currently allowed in the majority of jurisdictions to order imaging studies, clinical suspicion of an SRE often leads to delays in initiating important imaging tests that could assist in the diagnostic process. More research is warranted to explore the utilization of PTs and the integration of imaging into routine acute care practice.

## Conclusion

Although imaging indicated a femoral metastatic lesion and the patient reported hip symptoms, no initial restrictions or weight-bearing modifications were initiated for this patient. Upon recognition of increasing pain with weight bearing and functional limitations by the PT, collaboration with the interdisciplinary team followed, resulting in surgical stabilization of the hip. This case provides evidence of the critical role of PTs as movement specialists in advocating for their patients, especially in the areas of concern. As many hospitalized cancer patients have metastatic bone disease, routine surveillance for potential SREs should be standard practice to ensure optimal outcomes and patient safety.

## Data Availability

The original contributions presented in the study are included in the article/Supplementary Material; further inquiries can be directed to the corresponding author.
